# The combined application of salt-alkali tolerant phosphate solubilizing microorganisms and phosphogypsum is an excellent measure for the future improvement of saline-alkali soils

**DOI:** 10.3389/fmicb.2024.1364487

**Published:** 2024-02-23

**Authors:** Lingli Li, Shiqi Yang, Xin Hu, Zhen Li, Haoming Chen

**Affiliations:** ^1^School of Environmental and Biological Engineering, Nanjing University of Science and Technology, Nanjing, China; ^2^College of Resources and Environmental Sciences, Nanjing Agricultural University, Nanjing, Jiangsu, China

**Keywords:** microbial remediation, salt and alkali stress, co-remediation, phosphogypsum, organic acids, dissolution and release

## 1 Introduction

### 1.1 Saline-alkali soils not only affect food security but also hinder human social development

Soil salinization has always been a major threat to the sustainable development of agriculture and the improvement of land use efficiency (Meena et al., 2019; Kumawat et al., 2022). Salt-alkali soil, characterized by the accumulation of salt in the surface layer and an excessively high pH level, can be broadly categorized into three types: saline-alkali soil, alkaline soil, and saline-alkali soil. When the soil salt ion content is higher than 0.1%, the soil pH is higher than 8.0 or the sodium alkalinity is higher than 5%, it can be called saline-alkali soil. In nature, soil salinization and alkalization often occur simultaneously. Soil salinization is divided into primary salinization and secondary salinization. Primary salinization is mostly caused by climate, hydrology, and topography, while secondary salinization is mostly caused by the overuse of fertilizers and pesticides as well as irrational irrigation methods (Negacz et al., 2022). High salinity and elevated pH levels exert multifaceted negative impacts on soil. Firstly, salt ions attract and immobilize soil particles, resulting in soil compaction and decreased porosity, which in turn hinders the exchange of water, air, and nutrients with plant roots. Additionally, high salinity diminishes the soil's capacity to absorb beneficial nutrients. Secondly, elevated pH levels cause cations to bind to soil particles, further contributing to soil compaction and affecting the availability of micronutrients as well as organic matter decomposition. Ultimately, these negative impacts severely constrain soil fertility and plant growth. Currently, more than 100 countries around the world are affected by soil salinization. The most severe soil salinization is primarily found in regions such as North, East, and Southern Africa, the western United States, the Middle East, Central Asia, western China, the Yellow River basin, as well as Australia (Li et al., 2019; Negacz et al., 2022). The global map of saline-alkali soils in 2021 shows that 20–50% of irrigated soils in all continents have excessive salinity (FAO, 2021), which means that more than 1.5 billion people around the world are facing significant challenges in food production due to soil degradation. Moreover, about 1.5 million hectares of irrigated land are rendered unfit for cultivation due to severe salinization every year (Dey et al., 2021). Hence, the urgent need arises for the improvement and utilization of saline-alkali soils, given their significance as a crucial reserve land resource for food production and ecological environment construction.

### 1.2 Difficulty in utilizing phosphorus (P) is a non-negligible problem in soil salinization

The negative impact of salinization on soil is multifaceted. Firstly, the high salt content and high pH value of saline-alkali soil lead to soil hardening, reducing the soil's porosity and nutrient retention capacity (Negacz et al., 2022). Additionally, soil salinization also reduces the activity of soil microorganisms, affecting soil respiration and the activity of various enzymes such as alkaline phosphatase, urease, and catalase (Kumawat et al., 2022). In terms of plant growth, the high osmotic pressure of saline-alkali soil can interfere with the water absorption capacity of root cells, leading to cell dehydration and thus affecting the normal growth of plants. Additionally, saline-alkali soil can also increase plant uptake of soluble salt ions, which may have a negative impact on the material stability within plant cells, reducing photosynthetic rates and leading to plant wilting or even death (Parida and Das, 2005). More in-depth research has shown that increased salinity can lead to a series of problems such as ion toxicity, nutrient limitation, high osmotic stress, and oxidative stress. These problems can cause serious damage to processes such as enzyme activity, DNA, RNA, cell division, and protein synthesis in plants (Zhang et al., 2015; Kumawat et al., 2022).

The issue of P immobilization in saline-alkali soils should be given high priority because P is an essential element for plant and microbial growth. Although the total P content in some saline-alkali soils is relatively high, most of the P (60–90%) is fixed in the form of cations such as calcium phosphate, magnesium phosphate, aluminum phosphate, and iron phosphate (Jiang et al., 2019; Dey et al., 2021). Moreover, phosphorus fertilizers applied to saline-alkali soils tend to be preferentially fixed in the inorganic phosphorus pool, rather than being directly utilized by plants. To mitigate these effects, further research is needed to investigate how to improve the utilization efficiency of P in soil and mitigate the negative impact of salinization on plant growth by improving soil conditions.

## 2 Microbial remediation and mineral amelioration have received much attention in the remediation of saline-alkali soils

Currently, scholars at home and abroad have proposed a series of improvement measures for saline-alkali soil, including water conservancy, physics, chemistry, and biology. Physical improvement is usually achieved by reducing soil salinity, mainly based on the principles of water and salt movement. Basic methods include soil leaching, which involves the dissolution of salts in saline-alkali soils through freshwater irrigation. The resulting salt components are then transported to deeper soil layers through infiltration or drained away through drainage measures. These methods can also be classified as water conservancy engineering restoration technologies (Kumawat et al., 2022). The chemical improvement method for saline-alkali soil is mainly achieved by adding chemical reagents that react with salt-alkali ions (mainly Na^+^) in the soil. This reduces the content of soil salt-alkali components and improves the physical and chemical properties and soil structure. Commonly used chemical reagents include gypsum/phosphogypsum, humic acid, superphosphate, peat, and vinegar residue, etc. The biological improvement method (mainly plants and microorganisms) can essentially improve the physical and chemical properties of soil, while increasing soil fertility. It is a technology that integrates economic, environmental, and ecological effects. Among them, soil microorganisms are the most dynamic component of soil, affecting soil energy flow and material circulation through metabolic activities. Many salt-tolerant and alkali-tolerant microorganisms can not only reduce soil salinity, but also have the functions of nitrogen fixation, increasing potassium and phosphate dissolution. Meanwhile, these microorganisms secrete active substances, including plant hormones, iron carriers, antioxidants, and extracellular polysaccharides, which can activate the antioxidant enzyme system in plants and promote their growth. Compared to conventional physical and chemical improvement or remediation methods, in agricultural soil improvement, the choice of technology needs to be very cautious, which also leads to many physical and chemical improvement methods being rejected due to cost, environmental protection, etc. In current research, microbial remediation of saline-alkali soil has occupied a major position in most countries. Therefore, the introduction of salt-tolerant functional microorganisms can bolster the remediation of saline-alkali soils and augment plant resistance to salt stress.

## 3 The difficulty of P utilization in saline-alkali soils gives a place to phosphate solubilizing microorganisms (PSMs)

PSMs are a type of soil functional microorganism that was first discovered in the roots of farmland crops (Dey et al., 2021). It showed that in the community of salt-tolerant microorganisms, most species of PSMs are considered to be key plant growth-promoting microorganisms, which have the ability to solubilize P and K, and produce various metabolites that promote plant growth under saline conditions (such as plant growth hormones, iron carriers, ACC deaminase, and antagonists of plant pathogens) (Su et al., 2023). The most beneficial function of PSMs is to convert non-biologically available sources of P (both organic and inorganic) in soil into bio-available forms: 1. solubilization of inorganic P salts through cell-produced biological functions such as protonation, acidification, chelation, etc. 2. mineralization of organic P salts through enzyme activities (such as phosphatase, phosphonate hydrolase, phytases, and C-P lyase) (Ahemad, 2015; Hu and Chen, 2023). However, we must recognize that high salt concentration environments can lead to cell dehydration, shrinkage, and loss of activity (Rath and Rousk, 2015). Therefore, in order to improve the effect of microorganism's application in saline-alkali soil, it is necessary to screen out and select highly tolerant PSMs in saline-alkali soil during the process, and to use other technologies to maintain the activity of PSMs and their effect.

## 4 Phosphogypsum is a low-cost material for ameliorating saline-alkali soils

The application of gypsum in improving saline-alkali soil has been widely recognized, and the key lies in effectively reducing soil salt content through the displacement of Ca^2+^ and Na^+^ (Basak et al., 2022). Non-renewable natural gypsum minerals pose challenges for large-scale agricultural use. Meanwhile, due to industrial growth, government subsidies have decreased (especially in developing countries), and the quality of gypsum raw materials has decreased, leading to high costs of chemical remediation and further impeding the repair of saline-alkali soils (Qadir and Oster, 2004).

With the rapid development of industry, the production of gypsum and its similar by-products (such as PG and desulfurization gypsum) is gradually increasing, and the main component of these by-products is CaSO_4_ · 2H_2_O. PG is a solid waste from the wet phosphoric acid process, and its treatment is difficult. It is usually only stored or discarded in a centralized manner. The environmental hazards of PG mainly come from the release of P and heavy metals that are enriched in the soil or water body after large-scale accumulation, leading to high local concentrations and ecological damage. However, with the progress of process technology and the implementation of relevant policies, the content of polluting elements in PG produced in industrial production has been significantly reduced. For example, the weight percentage of heavy metals such as Pb, Cd and Cr in the PG used in the study of Chen et al. (2021) was ≤0.002 wt%. Additionally, countries such as Brazil, Spain and Lebanon have widely used PG as a soil amendment (Abril et al., 2009; Kassir et al., 2011; Costa et al., 2021).

Besides reducing salinity by replacing Na^+^, PG can also improve soil pore structure, increase water permeability, reduce soil redox potential (reduce negative values), and reduce CH_4_ emissions in saline-alkali soils (Khatun et al., 2021). Armstrong (1989) compared the solubility, exchangeable sodium replacement, and clay dispersion inhibition of three calcium amendments (PG, rock gypsum, saturated gypsum solution), and the results showed that PG was the most effective in saline-alkali soil improvement. Additionally, the total P content in PG is about 2.5–7.5%, and its reuse as a P fertilizer can reduce the damage to the environment and natural resources caused by P mining. However, under alkaline soil conditions, the application of PG can lead to the precipitation of Ca–P, thereby limiting the utilization efficiency of P in PG.

## 5 The combination of PSMs and PG for saline-alkali soil improvement is a measure for resource utilization

It is gratifying that the utilization of PSMs to solubilize insoluble P minerals and enhance P release has been proven to be feasible. The secretion of multiple organic acids makes PSMs more suitable for working with mineral soil adsorbents than other types of plant growth-promoting bacteria. For instance, the PSMs (*Burkholderia* sp. strain PH10) can completely dissolve apatite crystals within 22 h (Fontaine et al., 2016). The phosphate-solubilizing *B. megaterium* (TBRC 1396) can solubilize up to 835.45 ± 11.76 mg/L phosphorus from struvite in 14 days (Jokkaew et al., 2022).

**Figure 1:**
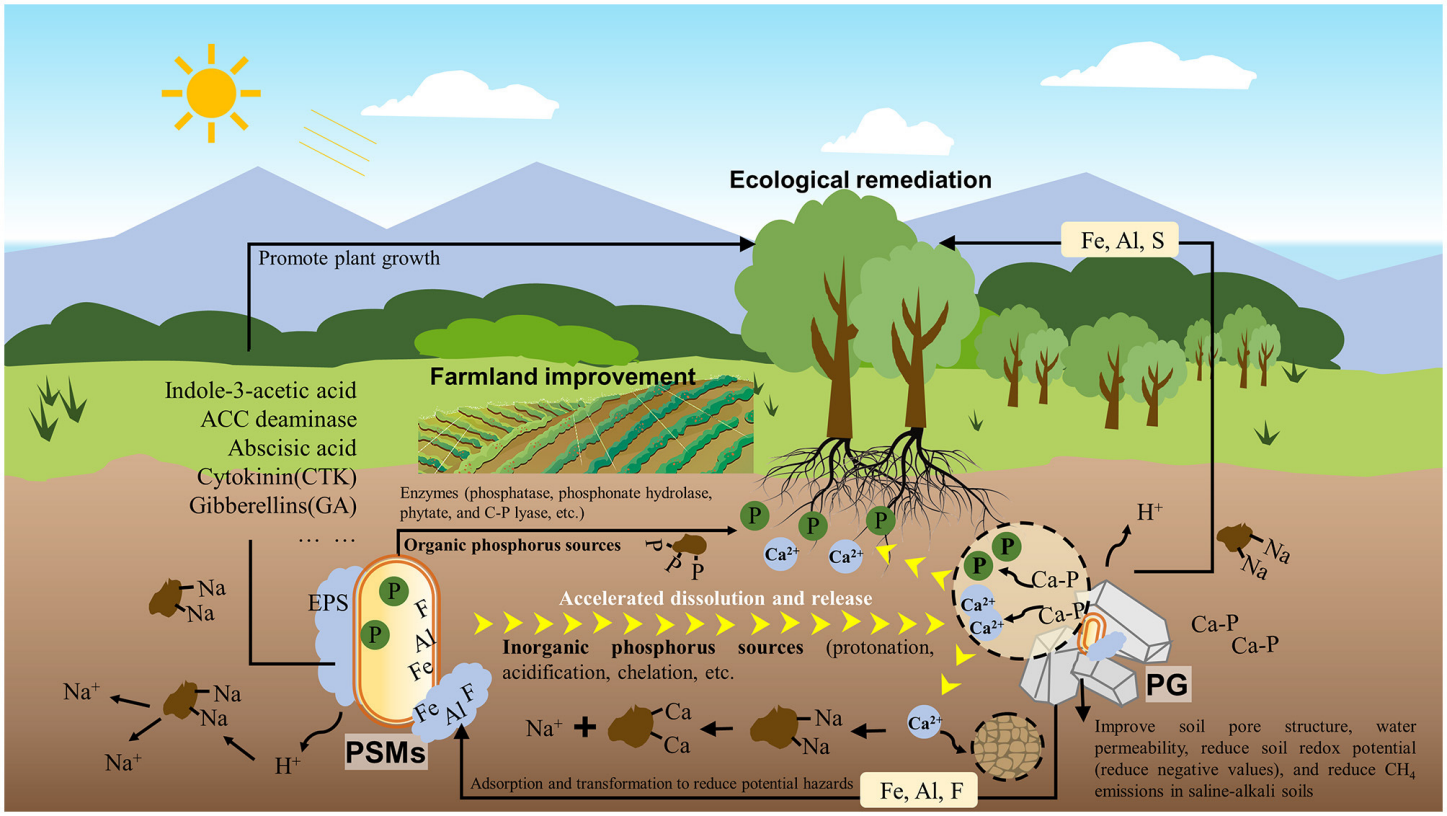
Phosphate solubilizing microorganisms (PSMs) combined with phosphogypsum (PG) can effectively repair saline-alkali soil remediation.

Multiple studies have confirmed that oxalic acid, citric acid, acetic acid, lactic acid, gluconic acid, and malic acid, which are organic acids secreted by PSMs, are the main means for dissolving inorganic insoluble phosphorus sources because these organic acids have strong acidity. Tian et al. (2022) found that *Aspergillus niger* (ANG) released up to 1,103 mg/L P from PG in 7 days. In addition, the rich P, Ca and organic matter in PG have been confirmed to be useful as fertilizers to promote plant growth. For example, adding 30 g/kg PG and a mixed microbial agent containing phosphate-solubilizing bacteria *Bacillus megaterium* var. phosphaticum and *Pseudomonas fluorescens* to the soil can increase the available phosphorus in the soil by ~80%, and increase the dry weight of corn (mg per plant) from 398 to 624 (Al-Enazy et al., 2017). Meanwhile, the application of PG (9 t ha^−1^) and bacteria (*A. lipoferum* + *B. circulance*) can significantly improve the physiological status, antioxidant enzyme activity, microbial activity, nutrient absorption, and productivity of maize plants under saline-sodic soil conditions (Khalifa et al., 2021). It is worth noting that the combination of microorganisms and PG can also reduce potentially harmful elements in PG, such as Fe, Al, F, etc., through their own cells and secretions (such as, extracellular polymers) (Jalali et al., 2016; Chen et al., 2021). Therefore, the combined application of PSMs and PG for saline-alkali soil remediation is feasible and effective, as the combination of the two not only leverages their respective advantages, but also leads to mutual enhancement (Figure 1).

## 6 Prospects for the combined application of PSMs and PG

The improvement of saline-alkali soil with PSMs and PG, and the promotion of plant growth in saline-alkali soil, is a potentially efficient and economic measure in the future. However, when trying to implement both technologies simultaneously in practical engineering applications, we should consider the following aspects. Firstly, attention should be paid to microbial diversity, and the beneficial microbial communities should be protected and utilized to achieve comprehensive soil improvement and ecological restoration. Secondly, the utilization of waste PG should be optimized (including raw material composition, usage amount, application frequency, application process, etc.) to reduce its negative impact on soil and the environment, while exploring its potential phosphorus resource value. Thirdly, long-term effect evaluation should be emphasized, and the impact of improvement measures on soil physical and chemical properties, microbial community structure, crop growth, and other aspects should be continuously monitored to ensure the stability and sustainability of the improvement measures. Fourth, emphasis should be placed on assessing environmental risks, particularly the damage and residual effects of radioactive elements on plants, to ensure that the improvement measures do not have negative impacts on the environment and human health. Finally, attention should be paid to technological innovation and application, exploring new composite improvement technologies and methods, and improving the utilization rate and improvement effect of PSMs and PG resources, thus making greater contributions to agricultural sustainable development and ecological environmental protection.

## Author contributions

LL: Data curation, Formal analysis, Writing – original draft. SY: Data curation, Methodology, Writing – original draft. XH: Data curation, Methodology, Writing – original draft. ZL: Methodology, Writing – review & editing. HC: Writing – original draft, Writing – review & editing.
